# Pulmonary Toxicity Assessment after a Single Intratracheal Inhalation of Chlorhexidine Aerosol in Mice

**DOI:** 10.3390/toxics11110910

**Published:** 2023-11-07

**Authors:** Jianzhong Zhang, Xinmin Jiang, Xin Li, He Sun, Mingyue Wang, Wanjun Zhang, Haonan Li, Hongmei Wang, Min Zhuang, Lin Zhang, Lin Lu, Jinglong Tang

**Affiliations:** 1 Shandong Provincial Maternal and Child Health Care Hospital Affiliated to Qingdao University, Jinan 250001, China; zydf12345@gmail.com (J.Z.); xm3083630017@163.com (X.J.); zhanglin8901@sdu.edu.cn (L.Z.); 2 Department of Environmental and Occupational Health, School of Public Health, Qingdao University, Qingdao 266071, China; lixin2023@nanoctr.cn (X.L.); sunhe9992021@163.com (H.S.); wmy2021@163.com (M.W.); zhangwanjun0130@163.com (W.Z.); 18209519394@163.com (H.L.); 3 Department of Respiratory Medicine, Affiliated Hospital of Medical College of Qingdao University, Qingdao 266021, China; dor.whm@163.com (H.W.); qyzhuangmin@126.com (M.Z.)

**Keywords:** chlorhexidine, toxicity, inhalation exposure, lung injury, transcriptome

## Abstract

Guanidine disinfectants are important chemical agents with a broad spectrum of activity that are effective against most microorganisms. Chlorhexidine, one of the most used guanidine disinfectants, is added to shampoo and mouthwash and applied in medical device sterilization. During the use of chlorhexidine, aerosols with micron particle size may be formed, which may cause inhalation toxicity. To assess the toxicity of inhaled chlorhexidine aerosol, mice underwent the intratracheal instillation of different concentrations of chlorhexidine (0, 0.125%, 0.25%, 0.5%, and 1%) using a MicroSprayer Aerosolizer. The mice were exposed for eight weeks and then sacrificed to obtain lung tissue for subsequent experiments. Histopathology staining revealed damaged lung tissues and increased collagen exudation. At the same time, pulmonary function tests showed that chlorhexidine exposure could cause restrictive ventilatory dysfunction, consistent with pulmonary fibrosis. The results of transcriptome analyses suggest that chlorhexidine may trigger an inflammatory response and promote the activation of pathways related to extracellular matrix deposition. Further, we identified that chlorhexidine exposure might enhance mucus secretion by up-regulating *Muc5b* and *Muc5ac* genes, thereby inducing fibrosis-like injury. These findings underscore the need for standardized use of disinfectants and the assessment of their inhalation toxicity.

## 1. Introduction

In recent years, the use of disinfectants has seen a significant increase, with guanidine disinfectants emerging as a popular choice for home sterilization and disinfection. These disinfectants are primarily composed of chlorhexidine and polyhexamethylene guanidine (PHMG)-type disinfectants. Guanidine disinfectants destroy bacterial cell membranes and exert antibacterial effects mainly through the cationic charge contained in the guanidine structure [1]. They are widely used in swimming pool treatment, paint fabric softeners, plastics, and preservatives, and were once considered harmless to humans [2]. A public health incident of lung injury caused by the aerosol inhalation of humidifier disinfectant (mainly PHMG) in South Korea began to make people aware of the inhalation toxicity of guanidine disinfectants [3,4,5]. The Scientific Committee on Consumer Safety (SCCS) stipulates that polyhexamethylene biguanidine (PHMB) may no longer be used as an antiseptic or for disinfection, other than in food, feed, cosmetics, pharmaceutical products, or medical devices, but not for the disinfection of drinking water for humans and animals, due to its potential toxicity. However, chlorhexidine has not been restricted as a kind of guanidine disinfectant. There are no systematic research data on or safety standards regarding chlorhexidine’s respiratory toxicity, its related products’ respiratory toxicities are not clearly marked or labeled, and there is a lack of safety warnings. Furthermore, the global market for chlorhexidine gluconate is expected to increase from USD 174.9 million in 2020 to USD 202.6 million in 2028. Given the escalating production of chlorhexidine, addressing the health risks linked to both occupational and non-occupational exposure to chlorhexidine, particularly inhalation exposure in the aerosol form, is of utmost importance.

Chlorhexidine, which has two biguanide groups, is a cationic disinfectant that destroys microbial cell membranes and induces the deposition of cell contents, thereby exerting antibacterial activity. It is widely used in medical, oral, and personal cleaning products in North America and Europe. Preoperative disinfection with 4% chlorhexidine has been demonstrated to reduce the risk of surgical site infection [6]. Various concentrations of chlorhexidine-containing mouthwash and nasopharyngeal soap are also used in cancer patients to decrease the incidence of respiratory tract infection after cancer surgery [7]. In addition, chlorhexidine is also used as a spray for the anti-inflammatory and antibacterial treatment of skin tissue [8]. However, the excessive and incorrect use of chlorhexidine may pose a potential risk of inhalation. During the use of chlorhexidine, aerosols with micron particle size may be formed, which may cause inhalation toxicity [9,10]. Chlorhexidine has been shown to have the potential to cause fibrosis. Previous studies have revealed that chlorhexidine dissolved in ethanol may cause peritonitis and peritoneal sclerosis in patients undergoing peritoneal dialysis [11]. Chlorhexidine is also commonly employed as a model to induce peritoneal fibrosis [12,13]. There are abundant studies on the inhalation toxicity of PHMG, but few studies on the toxicity of chlorhexidine as a guanidine disinfectant. The intratracheal instillation of chlorhexidine has been reported to cause lung tissue damage in rats and causes them to present symptoms like acute respiratory distress syndrome (ARDS) [14,15]. Nonetheless, the pulmonary toxicity and potential mechanisms of chlorhexidine aerosol inhalation exposure are yet to be fully understood.

Transcriptome analysis has become an essential tool for exploring toxicity and has proven effective in chemical risk assessment and ecotoxicological mechanism research. Furthermore, transcriptomics analyses can effectively compare the toxic effects of different toxicants, better understand the mechanisms of toxicity, and identify possible markers of toxicity and potential therapeutic targets. In this study, we established a mouse model of inhalation exposure to chlorhexidine aerosol and evaluated the inhalation toxicity of chlorhexidine aerosol via histopathological observation and lung function tests. Further, we utilized transcriptome analysis to identify differentially expressed genes, providing insight into the mechanism of lung injury induced through varying doses of chlorhexidine aerosol exposure and identifying potential biomarkers.

## 2. Materials and Methods

### 2.1. Animals and Exposure

Six- to eight-week-old C57BL/6J mice were procured from SPF (Beijing, China) Biotechnology Co., Ltd., and housed in a strictly controlled environment with five mice per cage, provided with clean water and ample food. The mice were maintained at an ambient temperature between 19 °C and 25 °C with a relative air humidity of approximately 40–60%. They were given 12 h of light and 12 h of darkness daily. A 7-day acclimatization period was provided before the start of animal experiments to ensure their adaptation to the new environment. The experimental procedures in this study were approved by the Animal Experimentation Ethics Committee of Qingdao University and followed the guidelines of the Guide for the Care and Use of Laboratory Animals.

**Figure 1:**
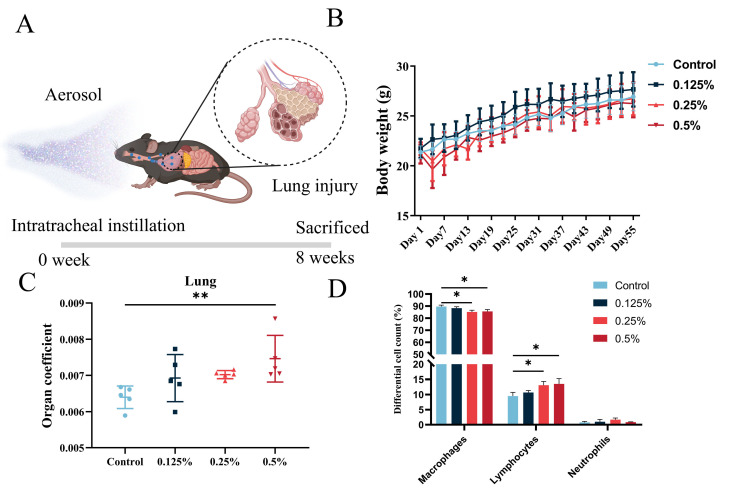
General toxicological evaluation of inhaled exposure to chlorhexidine aerosol and pulmonary immune response. (**A**) Schematic diagram of inhalation exposure model of chlorhexidine aerosol. (**B**) The body weights of mice in different dose groups (at least 12 mice in each group). (**C**) The organ coefficient of lung tissue in different dose groups (*n* = 5). (**D**) Composition of cell populations in BALF (*n* = 3). Data were analyzed via one-way ANOVA with Dunnett’s post hoc test (* *p* < 0.05, ** *p* < 0.01).

Chlorhexidine gluconate (CHG, 19–21% aqueous solution, CAS No. 18472-51-0) was obtained from Macklin Biotechnology Co., Ltd. (Shanghai, China). To determine the safety dose of chlorhexidine aerosol inhalation, we divided mice into five groups who received different doses: 0% (0 mg/kg), 0.125% (2.5 mg/kg), 0.25% (5 mg/kg), 0.5% (10 mg/kg), and 1% (20 mg/kg), and the dilution process was carried out using RO water. Under isoflurane anesthesia, the mice were given a single intratracheal instillation of 50 μL CHG solution or RO water (Figure 1A) using a MicroSprayer Aerosolizer (Beijing Huironghe Technology Co., Ltd.; Beijing, China) and were sacrificed 8 weeks later. The control group was administered the same volume of RO water. The mice were kept warm with an electric blanket until they regained consciousness, and their survival rate was recorded within 24 h after exposure. BMDS (2.7, EPA, Washington, DC, USA) was used to calculate the benchmark dose (BMD) and the benchmark dose lower confidence limit (BMDL) for mortality. The surviving mice were monitored for eight weeks, and their body weight was measured every three days. After eight weeks of tracheal drip, the mice were euthanized using sodium pentobarbital, and their lungs, hearts, spleens, kidneys, and brains were collected and weighed to calculate the organ coefficients. By weighing mice’s lung tissue and body weight, we calculated the ratio of lung tissue to body weight to obtain the lung organ coefficient. Some organs were fixed in 4% paraformaldehyde (Biosharp, Beijing, China) for histological analysis, while others were snap-frozen in liquid nitrogen and stored at −80 °C for further research.

### 2.2. Bronchoalveolar Lavage Fluid

Three mice in each group were randomly selected for bronchoalveolar lavage. Firstly, an indwelling needle was used to intubate the anesthetized mice. The mice were lavaged three times with a 1.0 mL syringe and cold Dulbecco phosphate-buffered saline (DPBS, PB180329, Procell, Wuhan, China). Secondly, the collected bronchoalveolar lavage fluid (BALF) was centrifuged at 4 °C with a rotational speed of 1000 g and time of 10 min. After centrifugation, the supernatant was removed, the residues collected were resuspended with 50 μL DPBS, and the cells were smeared onto the slides. After static drying, the inflammatory cells were stained using the Diff-Quick Stain Kit (Yeasen, Shanghai, China). The macrophages, lymphocytes, and neutrophils in the slide were counted using an oil mirror, and 400 cells were counted in each slide.

### 2.3. Histopathological Analysis of Mouse Tissues

Samples were taken from a 4% paraformaldehyde solution to examine histopathological changes. The lung tissue was initially embedded in paraffin and then sectioned continuously at a thickness of 4 μm. The sections were stained using H&E and Masson, dehydrated with anhydrous ethanol, and sealed with neutral gum. Three mice were selected randomly from each group, and five images of each mouse were taken randomly to calculate the Mikawa score. The Mikawa score was determined based on capillary congestion, alveolar fibrin exudation, neutrophil exudation, the exfoliation of airway mucosal epithelial cells, and the widening of the alveolar septum [16]. The scores were tallied to estimate the severity of the lung tissue injury. The results of pathological staining were photographed and analyzed using an orthogonal microscope. Pathological sections from three mice were analyzed in each group, with five randomly selected fields of view photographed for the microscopic examination of each section, and collagen fibril exudation in mouse lung tissue was measured using Image J software (https://imagej.net/) (Media Cybernetics, Silver Spring, Washington, DC, USA).

### 2.4. Pulmonary Function Test

After anesthesia with pentobarbital sodium, mice were intubated and subjected to pulmonary function tests [17] using the FlexVent FX system (SCIREQ, Montreal, QC, Canada), with test procedures and score calculations strictly adhering to the manufacturer’s guidelines. The system comprises two modules, FX1 and NPFE, and utilizes FlexiVent 8.0 for calculations. During testing, the tidal volume was set at 10 mL/kg, the respiratory rate at 150 bpm, the inspiratory–expiratory ratio at 2:3, and the positive end expiratory pressure at 3 cmH_2_O. To equalize alveolar and external forces, the mouse lungs were inflated for three seconds at the start of the test, achieving an intrapulmonary pressure of 30 cmH_2_O, and the volume difference between the beginning and end was used to determine the deep inspiratory volume (IC), which refers to the maximum amount of air that can be inhaled vigorously at the end of calm exhalation. The single frequency concussion mode Snapshot measured respiratory system compliance (Crs), respiratory system resistance (Rrs), and respiratory system elastance (Ers). In contrast, the volume-driven concussion Prime mode recorded volume and pressure signals converted into velocity signals to reflect central airway resistance (Rn, reflects the airway resistance of the large airway without the small airway involved in gas exchange), tissue elastance (H, represents the elastic potential energy stored in the tissue), tissue damping (G, related to tissue resistance and also includes part of the peripheral airway resistance), and other indicators of mouse respiratory function. In addition, the NPFE model was utilized to obtain forced vital capacity (FVC), forced expiratory volume at 0.1 s (FEV_0.1_), FEV_0.1_/FVC, and peak expiratory flows (PEF).

### 2.5. Transcriptome Analysis

Total RNA was extracted from lung tissues using the TRIzol LS reagent (Invitrogen, Carlsbad, CA, USA) following the manufacturer’s protocol. Cleaved RNA fragments were reverse-transcribed using SuperScriptTM II Reverse Transcriptase (Invitrogen, Carlsbad, CA, USA) to generate cDNA, which was then subjected to 2 × 150 bp paired-end sequencing (PE150) on an Illumina NovaseqTM 6000 at Biomark Technologies Co., Ltd. (Beijing, China), according to the recommended protocol. The differentially expressed genes (DEGs) were analyzed using Deseq2 (1.34.0), with genes exhibiting *FDR* < 0.05 and |LogFC| > 1 considered as DEGs. The DEGs were then subjected to Gene Ontology (GO) and Kyoto Encyclopedia of Genes and Genomes (KEGG) enrichment analysis using the R package ClusterProfiler (4.0.5) to identify significant functional pathways.

### 2.6. Real-Time Quantitative PCR Analysis

This study used the TRIzol reagent (Invitrogen, Carlsbad, CA, USA) to extract total RNA from frozen lung tissues, following the manufacturer’s instructions. Subsequently, the quantity and quality of the isolated total RNA were assessed using a NanoDrop ONE Spectrophotometer (NanoDrop Technologies, Wilmington, DE, USA). The obtained RNA was then subjected to reverse transcription using the HiScript^®^III RT SuperMix (Vazyme, Nanjing, China). The real-time quantitative PCR (qPCR) was performed using the ChamQ Universal SYBR qPCR Master Mix (Vazyme), with β-actin as an internal control. Supplementary Table S1 provides information on the primers used. All qPCR reactions were performed in triplicate, and fold changes in gene expression were calculated using the 2^−ΔΔCt^ technique. These exact steps were taken to ensure the accuracy and reproducibility of the data obtained.

### 2.7. Statistical Analysis

Data from all experiments in this research are given as means with standard deviations (SDs). GraphPad Prism 8.0 (Dotmatics) was used to create the graphics, and SPSS 22.0 (IBM, Armonk, NY, USA) was used to analyze the statistical data. The differences between the CHG exposure and control groups were compared using Student’s *t*-test or one-way ANOVA followed by Dunnett’s post hoc test (with *p* < 0.05 considered statistically significant).

## 3. Results

### 3.1. Benchmark Dose of Mice Mortality Rate after CHG Aerosol Inhalation

By administering CHG solutions of varying concentrations into the trachea of mice, we observed a clear dose-dependent relationship between CHG concentration and mouse mortality within 24 h (Table S2). Notably, mice exhibited no mortality when the intratracheal instillation concentration was 0% or 0.125%. However, at an instillation concentration of 0.25%, the mortality rate rose to 32.1% and increased to 42.9% at a concentration of 0.5%. Remarkably, when the drip dose was increased to 1%, mouse mortality was found to be as high as 70.0%. We calculated the BMD and BMDL to quantify the relationship between instillation dose and mouse mortality. Our analysis identified a monotonically increasing dose–response pattern. Of the eight models tested, the quantal linear model exhibited the lowest AIC value and the highest *p* value and was chosen as the best-fitted model to estimate the relationship between CHG concentration of single intratracheal instillation and mouse death (*p* = 0.628). Utilizing this model, we calculated that the BMDL of CHG aerosol inhalation-induced mouse death was 0.066% (1.32 mg/kg), as shown in Table 1.

### 3.2. General Toxicological Assessment of CHG Aerosol Inhalation

Following a single intratracheal instillation of CHG, we conducted an eight-week study in mice to assess the impact of CHG exposure on body weight and lung tissue. We measured the weight of the mice every three days during the feeding period and found that mice in the 0.25% and 0.5% instillation dose groups exhibited a temporary decrease in body weight within three days after tracheal instillation. However, their body weight returned to baseline levels and steadily increased one week later, as depicted in Figure 1B. At the end of the eight weeks, the mice were euthanized, and their lung tissue was weighed to obtain the lung organ coefficient. Our analysis revealed that the lung organ coefficient of mice in the highest exposure group was significantly higher than that of the control group, as shown in Figure 1C (*p* < 0.05). These findings suggest that respiratory exposure to CHG may cause abnormal changes in mice, such as pulmonary oedema or increased collagen exudation.

To assess whether respiratory exposure to CHG could induce lung inflammation, we analyzed mice’s inflammatory cells proportion in BALF. The inflammatory cells in the BALF were predominantly composed of macrophages, lymphocytes, and neutrophils. We quantified the number of these three types of inflammatory cells in the BALF and observed that, compared with the control group, the number of lymphocytes and neutrophils generally increased in the exposure group. Notably, the proportion of lymphocytes in each exposure group was found to be elevated by 1.17%, 3.60%, and 4.03%, respectively, and the ratio of lymphocytes in the middle and high dose groups was significantly higher (*p* < 0.05) than that in the control group (Figure 1D and Table S3). Our findings suggest that inhalation exposure to CHG may trigger pulmonary inflammatory reactions in mice.

### 3.3. CHG Aerosol Inhalation Triggered Lung Histopathological Changes and Collagen Deposition

**Figure 2:**
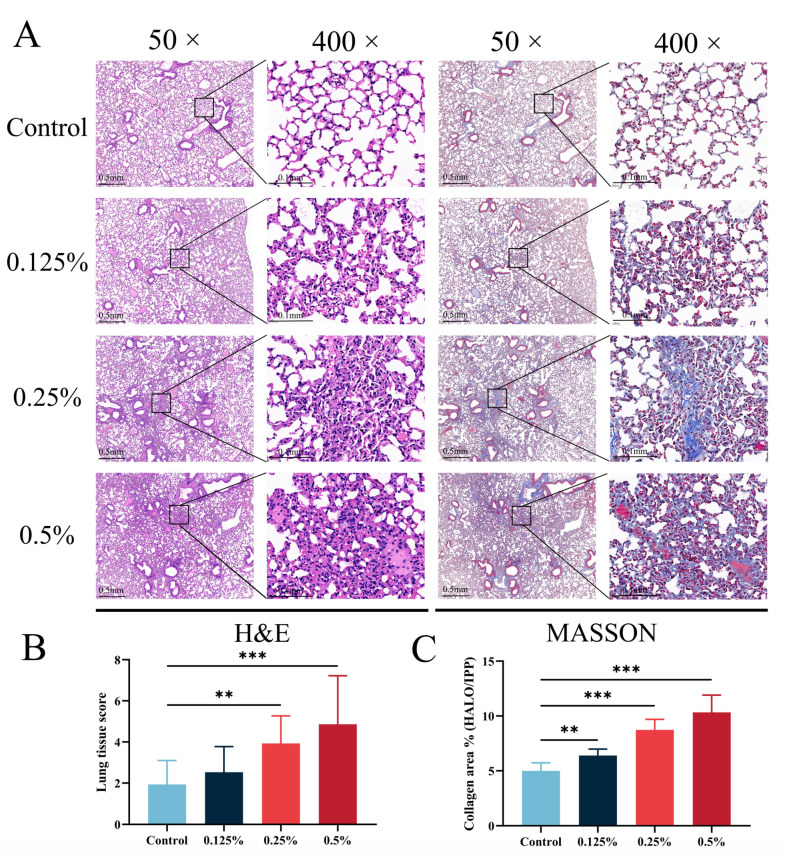
Chlorhexidine aerosol inhalation exposure caused lung histopathological changes and lung collagen deposition. (**A**) The lung sections stained with H&E and Masson (*n* = 3). (**B**) The results of lung tissue score (*n* = 3). (**C**) The percentage of collagen area in different groups (*n* = 3). Data were analyzed via one-way ANOVA with Dunnett’s post hoc test (** *p* < 0.01, *** *p* < 0.001).

To explore the impact of CHG inhalation exposure on lung tissue, we subjected lung tissue fixed with 4% paraformaldehyde to H&E and Masson staining (Figure 2A). H&E staining revealed that the alveolar structure in the control group was clear, and the lung parenchyma was normal. In contrast, the lung tissue of mice in the CHG exposure group displayed obvious damage that worsened with increasing exposure doses. The primary site of lung tissue injury occurred around the large airway, and the lung tissue showed marked signs of inflammatory cell infiltration, alveolar protein deposition, and airway epithelial cell exfoliation. Furthermore, we observed the thickening of the alveolar wall and alterations in the alveolar structure in the CHG exposure group, and the degree of these effects increased with higher exposure concentrations. To quantitatively evaluate the degree of lung tissue injury, we utilized the Mikawa score (Figure 2B). The degree of lung tissue injury in the exposure group displayed an upward trend gradient with increasing exposure concentration. Notably, there was a significant difference in the lung injury score between the middle and high-exposure dose group and the control group (*p* < 0.05).

Masson staining is a classical method of connective tissue staining that can provide a comprehensive assessment of collagen fiber deposition in tissue. In this study, we used Masson staining and quantitative analysis (Figure 2C) to examine the deposition of collagen fibers in the lung tissue of mice exposed to CHG and compared it to the control group. Our results showed that the exposed group exhibited a significantly larger area of collagen fiber deposition in the lung tissue, which increased with the exposure dose. Specifically, collagen fibers were found in the connective tissue surrounding the large airway, alveolar septum, stroma, and bronchi, accompanied by the thickening of the alveolar wall and deposition of protein. In areas where inflammatory cell infiltration was observed, a large amount of collagen fiber exudation was often present. The quantitative evaluation of the collagen area in lung tissue using Image J software showed a significant dose-dependent increase in collagen fiber deposition in the exposed group compared with the control group. The difference in collagen area between each dose group and the control group was statistically significant (*p* < 0.05). Furthermore, we examined the hydroxyproline content in the alveolar lavage fluid of CHG-exposed mice using ELISA (Figure S2). Consistent with the results of Masson staining, inhalation exposure to CHG increased hydroxyproline in mouse lung tissue. Our pathological staining and quantitative evaluation results indicate that inhalation exposure to CHG can cause lung injury in mice by inducing significant collagen fiber deposition in the lung tissue.

### 3.4. CHG Aerosol Inhalation Decreased Lung Function

**Figure 3:**
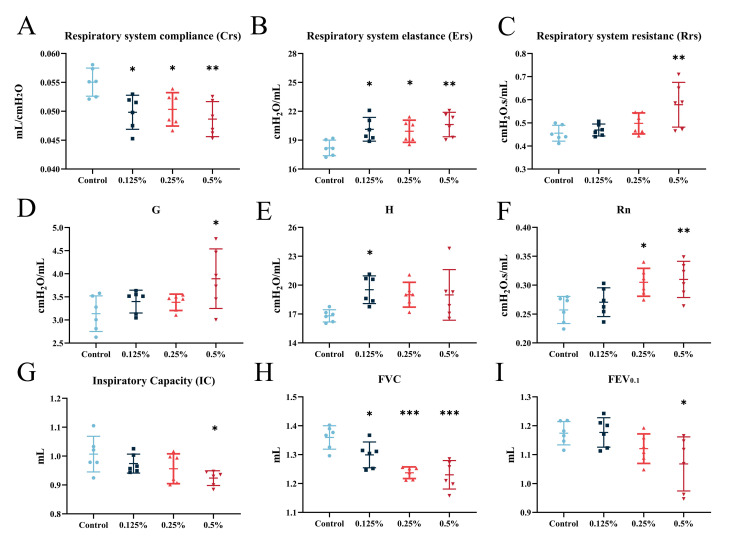
Chlorhexidine aerosol inhalation exposure decreased pulmonary function in mice. (**A**) Respiratory system compliance, (**B**) respiratory system elastance, (**C**) respiratory system resistance, (**D**) tissue damping, (**E**) tissue elastance, (**F**) central airway resistance, (**G**) inspiratory capacity, (**H**) forced vital capacity, and (**I**) forced expiratory volume at 0.1 s were measured in different groups using the flexiVent FX system. Data are presented as mean ± SD (*n* = six mice per group). Data were analyzed using one-way ANOVA with Dunnett’s post hoc test (* *p* < 0.05, ** *p* < 0.01, *** *p* < 0.001).

To investigate the impact of CHG respiratory exposure on respiratory function in mice, we employed the forced oscillation technique to measure pulmonary function in mice accurately (Figure 3). Our findings indicate that CHG aerosol exposure led to the total resistance of the respiratory system increasing, the elastic resistance increasing, and the compliance of lung decreasing (Figure 3A–C). This suggests that CHG aerosol exposure may increase the surface tension of lung tissue in mice. In Prime mode, we observed that CHG aerosol inhalation exposure worsened lung injury in mice (Figure 3D), consistent with the results obtained from H&E and Masson staining. The exposed group’s tissue elastance increased (Figure 3E), as confirmed by the increased Ers. Furthermore, respiratory exposure to CHG increased airway resistance in response to large airways (Figure 3F). In Deep Inflation mode, we found that the lung deep inspiratory capacity of mice exposed to CHG was significantly lower than that of control mice (Figure 3G). We also assessed pulmonary ventilation function using the NPFE model, often used to diagnose lung diseases. Our results showed that CHG inhalation exposure decreased FVC and FEV_0.1_ (reflecting ventilation function) in mice, but the FEV_0.1_/FVC has not changed significantly (Figure 3H,I and Figure S1A). PEF, a standard parameter reflecting lung inspiratory capacity and large airway function, also decreased after exposure (Figure S1B). Our study confirmed that inhalation exposure to CHG could lead to restrictive ventilatory dysfunction in mice.

### 3.5. Transcriptome Analysis of Mice Lungs after CHG Aerosol Inhalation

**Figure 4:**
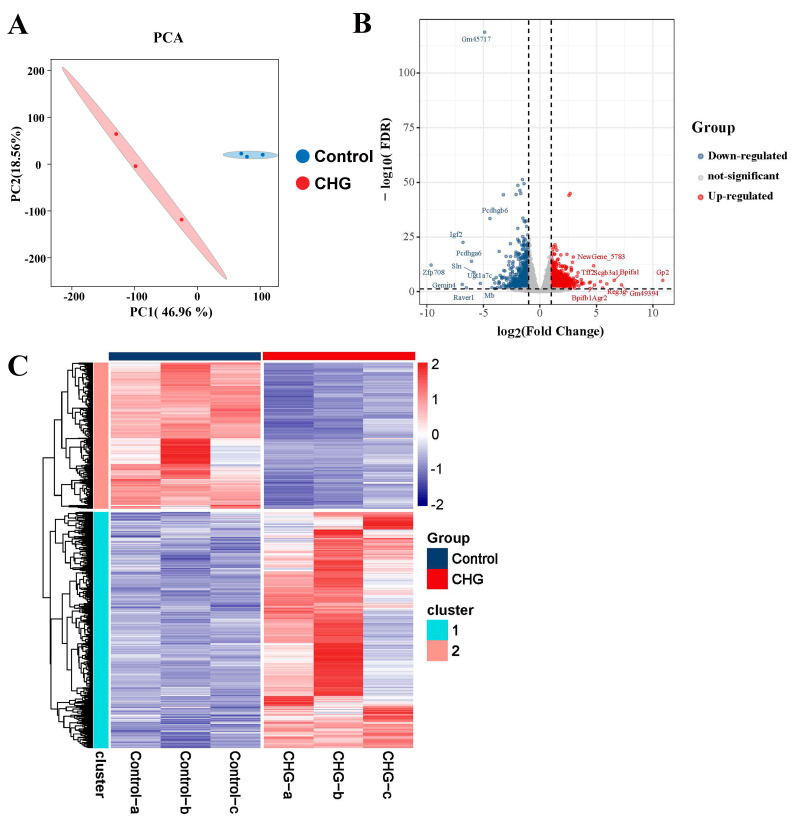
Differential expressed genes in lung tissue caused by chlorhexidine inhalation exposure. (**A**) PCA plot of the genes in all subjects (*n* = 3), Blue squares represent the control group and red squares represent the chlorhexidine group. (**B**) The volcano plot showing DEGs in 0.5% vs. control (*n* = 3). (**C**) Heatmap showing the DEGs between the control and the CHG exposure group (*n* = 3).

**Figure 5:**
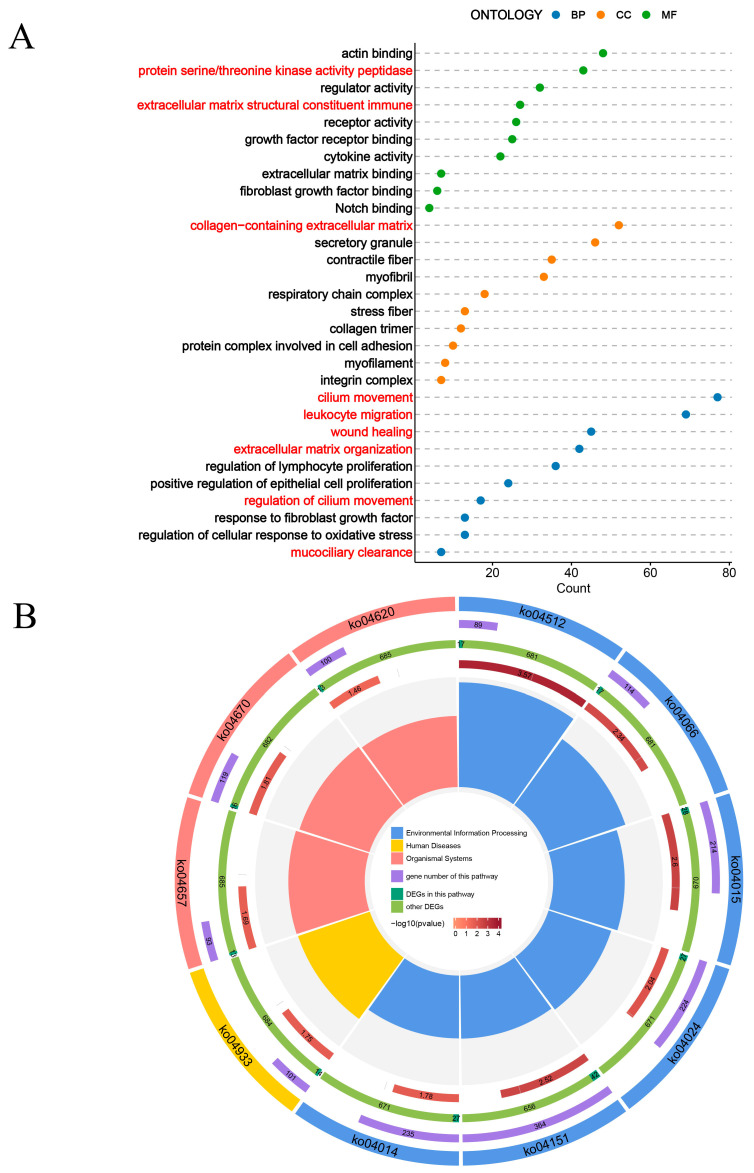
Functional enrichment analysis of DEGs after exposure to chlorhexidine. (**A**) The GO enrichment analysis in DEGs of 0.5% CHG vs. control (*n* = 3). (**B**) The KEGG enrichment analysis in DEGs of 0.5% CHG vs. control (*n* = 3).

To elucidate the underlying mechanism of lung injury caused by CHG inhalation, we conducted transcriptome sequencing of lung tissue from mice exposed to CHG (Figure 4). The PCA plot reflected the gene expression pattern of lung tissue after being treated with the CHG (Figure 4A). Compared with the control group, the transcriptional level of lung tissue in the CHG exposure group was significantly disturbed. The volcano plot and heatmap show DEGs in the CHG exposure group compared to the control group (Figure 4B,C). We observed that inhalation of 0.5% CHG led to 1998 DEGs, including 1235 up-regulated and 763 down-regulated genes. To further investigate the biological changes induced by CHG exposure, we conducted GO and KEGG enrichment analyses of DEGs (Figure 5). GO enrichment analysis revealed significant changes in pathways related to extracellular matrix organization and tissue remodeling, accompanied by the activation of certain mucociliary clearance-related pathways (Figure 5A). KEGG analysis revealed the considerable activation of pathways associated with leukocyte transendothelial migration (Ko04670), IL-17 signaling pathway (Ko04657), and PI3K-Akt signaling pathway (Ko04151) (Figure 5B). The activation of these pathways is closely related to the development of pulmonary fibrosis.

### 3.6. Explore and Verification of Essential Genes for Lung Fibrotic Injury Induced through CHG Aerosol Inhalation

**Figure 6:**
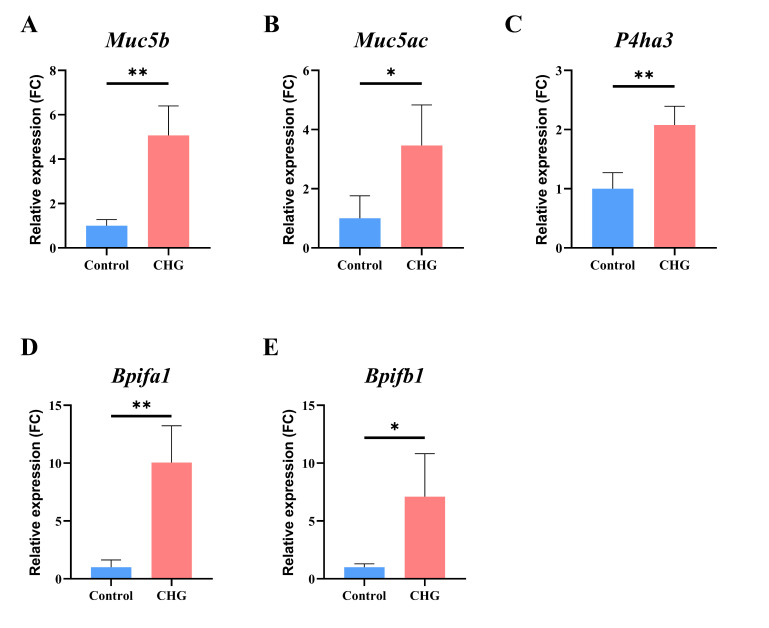
Differential expressed genes in the lung tissues of mice after exposure to chlorhexidine. The CHG group represents an intratracheal instillation concentration of 0.5%. Data presented are the average of three or four replicates. Data are shown as mean ± SD by qPCR (* *p* < 0.05, ** *p* < 0.01).

To verify the expression levels of essential genes, we used qPCR and observed that the expression levels of *Muc5ac*, *Muc5b*, *Bpifa1*, *Bpifb1*, and *P4ha3* were considerably higher compared to the control group (Figure 6). These findings provide valuable insights into the molecular mechanisms involved in the development of fibrosis-like lung injury after exposure to CHG through inhalation. Importantly, the changes observed in these genes using qPCR align with the results obtained from transcriptome sequencing, indicating the reliability of the transcriptome sequencing analysis.

## 4. Discussion

Considering the increased production and overuse of guanidine disinfectants, concerns have been raised regarding their impact on respiratory health. While disinfectant aerosols have become a popular choice for cleaning, limited knowledge exists regarding their potential adverse effects on the respiratory system. Addressing this gap, we present a study exploring the inhalation toxicity and latent mechanism of chlorhexidine aerosol. Employing the benchmark dose, we evaluated the acute toxicity of chlorhexidine inhalation and identified the evolution of pulmonary fibrosis as a significant and worrisome consequence of chlorhexidine-induced lung injury. Our transcriptome analysis further revealed that chlorhexidine aerosol inhalation could trigger immune activation and tissue remodeling in lung tissue, ultimately identifying genes responsible for driving lung injury. These findings provide valuable insight into the potential risk and biomarkers of chlorhexidine-induced lung injury, emphasizing the need to handle and use disinfectant aerosols appropriately.

Chlorhexidine is a symmetrical cationic molecule with two 4-chlorobenzene rings and two biguanide groups connected by a central six-membered ethylene chain, and has been widely used since the 1950s. While primarily administered orally or topically, its low bioavailability underestimates its toxicity during parenteral exposure. Like PHMG, chlorhexidine exerts a bactericidal effect by binding non-specifically to membrane phospholipids, destroying cell membranes [14]. However, studies have shown that chlorhexidine is significantly more cytotoxic than PHMB [18]. Additionally, in vitro studies have revealed the dose-dependent toxic effects of chlorhexidine on osteoblasts [19] and chondrocytes [20], as well as direct genotoxic and cytotoxic effects on lymphocytes and macrophages [21,22]. Reactive oxygen (ROS) production may be responsible for these effects. Acute damage to lung cell membranes caused by chlorhexidine may explain deaths from high-concentration drips. Previous studies show that the tracheal instillation of high concentrations of chlorhexidine led to extensive pulmonary dysfunction and rat deaths [14]. Furthermore, even when not inhaled directly, oral or intravenous administration can result in chlorhexidine accumulation in lung tissue [23]. Thus, the standardized daily and clinical application of chlorhexidine is necessary to prevent lung tissue injury resulting from multiple exposure pathways.

The histopathological examination of mouse lung tissue revealed that prolonged exposure to chlorhexidine aerosol could increase the coefficient of pulmonary inflammation and collagen deposition in mice, similar to pathological changes resembling pulmonary fibrosis induced by the aerosol inhalation of PHMG or the intratracheal instillation of bleomycin [24,25,26]. In addition, Xue et al. detected the blood routine and lung histopathology of 7-week-old Sprague Dawley rats via the intratracheal instillation of different concentrations of chlorhexidine solution [14]. The affected lung tissue displayed signs of alveolar and capillary congestion, marked edema, and the infiltration of inflammatory cells. In addition, other studies focused on acute respiratory distress syndrome (ARDS) caused by chlorhexidine [15,27,28]. The animal exposure experiments of 0.01%, 0.1%, and 1% chlorhexidine exposure showed that severe alveolar and pulmonary capillary congestion could be observed one day after chlorhexidine exposure. Similar to our results, tissue collagen exudation and inflammatory infiltration still exist after 7 days, 28 days, and 84 days of exposure. The exposure also affected the respiratory system’s mechanical properties, with significant decreases in IC, Crs, FVC, and FEV_0.1_, along with significant increases in Ers and Rrs. These findings suggest that chlorhexidine aerosol exposure can cause restrictive pulmonary ventilatory dysfunction, consistent with the pulmonary fibrosis induced by bleomycin and PHMG [24,29,30]. The alterations in pulmonary function parameters further support the notion of fibrosis-like changes, indicating that histopathological changes have impacted the mechanical properties of the lung and respiratory system.

Our analysis revealed that exposure to chlorhexidine aerosol could activate biological processes related to extracellular matrix deposition, which is a classic pathway of fibrosis. The main feature of fibrosis is the abnormal formation and remodeling of injury via fibrous connective tissue, accompanied by a large amount of extracellular matrix deposition [31]. Tissue injury is often accompanied by inflammation. Local immune cells are activated to produce a variety of biologically active cytokines and chemokines (such as the TGF-β family) [32]. These mediators lead to the local activation of mesenchymal cells, resulting in mesenchymal cell transformation and ECM deposition [33]. The inflammatory reaction induced by chlorhexidine aerosol may finally cause the deposition of ECM. *P4ha3*, as a target gene downstream of TGF-β, was significantly increased in the lung tissue of mice exposed to CHG. Its expression in the fibroblasts of bleomycin-induced fibrotic mice and patients with idiopathic pulmonary fibrosis increased significantly and was confirmed to be related to the increase in hydroxyproline [34]. In addition, we also found that cilia-related pathways were increased dramatically in the CHG exposure group. Aberrant ciliogenesis are found in the lung tissues of patients with usual interstitial pneumoniac (UIP)/idiopathic pulmonary fibrosis (IPF) and are closely related to the expression of *Muc5b* [35]. Genes related to mucus secretion, such as *Muc5b* and *Muc5ac*, were also significantly increased in the lung tissue of mice exposed to CHG. The overexpression of *Muc5b* may cause mucociliary dysfunction, hypoxia, and host defense changes; then promote the destruction of lung structure and the loss of local homeostasis; and eventually lead to fibrosis [36,37]. *Bpifa1* and *Bpifb1* are increased in cystic fibrosis and IPF [38,39], which is the most significantly increased secretome-associated protein in UIP [40]. Therefore, for the lung fibrosis-like pathological changes caused by long-term exposure to chlorhexidine, effective intervention on mucociliary clearance may be a better treatment.

This study investigated the lung tissue damage induced by a single intratracheal instillation of chlorhexidine and its underlying mechanisms. However, there are still some shortcomings in this study: firstly, only a single intratracheal instillation was performed in this study, and there is a lack of results related to lung tissue alterations induced by multiple exposures. Secondly, this study only examined lung tissue damage after 8 weeks of chlorhexidine infusion and lacked the experiments and discussion of early events. Finally, we only considered the injury effect of chlorhexidine on lung tissue. However, we did not consider the effect of chlorhexidine exposure on other tissues and organs. Therefore, further investigations may be needed to elucidate the inhalation toxicity of chlorhexidine exposure and its underlying mechanisms.

## 5. Conclusions

In summary, this study demonstrates that exposure to chlorhexidine aerosol can result in early fibrotic changes in lung tissue and a reduction in lung function. The results of transcriptome analyses suggest that chlorhexidine may trigger an inflammatory response and promote the activation of pathways related to extracellular matrix deposition, resulting in structural and functional changes in lung tissue. Furthermore, our findings indicate that chlorhexidine exposure may enhance mucus secretion by up-regulating the *Muc5b* and *Muc5ac* genes, thereby inducing fibrosis-like injury.

## Figures and Tables

**Table 1:** BMD and BMDLs of CHG inhalation dose and model fitness for mice mortality.

Model Name	BMD ^a^	BMDL ^b^	BMDU ^c^	*p*-Value	AIC ^d^
**Gamma**	0.112	0.067	0.202	0.433	93.635
**Logistic**	0.211	0.162	0.276	0.099	98.604
**Log-Logistic**	0.124	0.040	0.204	0.462	93.333
**Log-Probit**	0.135	0.050	0.209	0.485	93.052
**Multistage**	0.094	0.055	0.178	0.450	93.790
**Probit**	0.200	0.155	0.259	0.107	98.172
**Weibull**	0.106	0.067	0.192	0.438	93.689
**Quantal-Linear**	0.090	0.066	0.126	0.628	91.809

^a^ BMD, benchmark dose (concentration causes ten percent of mice to die); ^b^ BMDL, a lower bound of 95% confidence interval of BMD; ^c^ BMDU, an upper bound of 95% confidence interval of BMD; ^d^ AIC, Akaike information coefficient values; *p*-value, represents the global measurement of model fitness (goodness-of-fit, required > 0.1).

## Data Availability

The data can be obtained from corresponding author at reasonable request.

## Supplementary Materials

The following supporting information can be downloaded at: https://www.mdpi.com/article/10.3390/toxics11110910/s1, Table S1: Primer sequences for mouse samples used in qPCR (5′to 3′); Table S2: Mortality of chlorhexidine-exposed mice at different concentrations; Tabel S3: Percentage of inflammatory cells in bronchoalveolar lavage fluid. Figure S1: Chlorhexidine decreased pulmonary function in mice. (A) FVE0.1/FVC and (B) Peak expiratory flows (PEF) were measured using a NPFE model via the flexiVent FX system. Data were analyzed by one-way ANOVA with Dunnett’s post-hoc test (** *p* < 0.01); Figure S2: ELISA detection of hydroxyproline in BALF of control and CHG exposure groups. Data are presented as mean ± SD (*n* = 3, * *p* < 0.05).
