# Supplementary material for: Pulmonary Toxicity Assessment after a Single Intratracheal Inhalation of Chlorhexidine Aerosol in Mice
Source: Toxics. 2023 Nov 7;11(11):910. doi: 10.3390/toxics11110910 (PMC10675078; doi:10.3390/toxics11110910)
Supplement: Supplementary file 1 [file toxics-11-00910-s001.zip › toxics-2682234-supplementary.pdf]

Pulmonary Toxicity Assessment after a Single Intratracheal Inhalation of Chlorhexidine Aerosol in Mice

Jianzhong Zhang[1,2 †], Xinmin Jiang[1,2 †], Xin Li[2], He Sun[2], Mingyue Wang[2], Wanjun Zhang[2], Haonan Li[2], Hongmei Wang[3], Min Zhuang[3], Lin Zhang[1,2], Lin Lu[1,2 *], Jinglong Tang[1,2 *]

[1] Shandong Provincial Maternal and Child Health Care Hospital Affiliated to Qingdao University, Jinan 250001, China

[2] Department of Environmental and Occupational Health, School of Public Health, Qingdao University, Qingdao 266071, China

[3] Department of Respiratory Medicine, Affiliated Hospital of Medical College of Qingdao University, Qingdao 266021, China

[*] Correspondence: lulin163@126.com (L.L.); tangjinglong@qdu.edu.cn (J.T.)

[†] These authors contributed equally to this work

Summary of Supporting Information

Materials and methods:

ELISA

According to the protocol of the ELISA kit, the levels of Hydroxyproline in BALF of the control group and CHG exposure group were detected. Hydroxyproline (CSB-E08839m) ELISA kits were purchased from CUSABIO (Wuhan, https://www.cusabio.com/, China). Briefly, add 100 μl of standard and sample to be tested into the wells respectively, mix well, put on the plate sticker, incubate at 37 °C for two hours, discard the liquid in the wells, add 100 μl of biotin labelling antibody working solution, put on the plate sticker, incubate at 37 °C for one hour, discard the liquid in the wells. Wash three times with 200 μl washing solution, 2 minutes each, and shake dry. Add 100 μl of horseradish peroxidase labelled affinity protein working solution to each well, affix the plate sticker and incubate at 37 °C for one hour. Discard the liquid in the wells, wash with 200 μl of washing solution five times, two each time and shake dry. Add 90 μl of the substrate solution, develop the color at 37 °C for 15-30min, add 50 μl of termination solution, measure the optical density (OD) at 450 nm after 5 min, and calculate the concentration of each well. Samples from three animals per group were measured to derive the Mean ± SD.

Supplemental Tables and Figures:

Table S1. Primer sequences for mouse samples used in qPCR (5′to 3′).

Gene	Forward Primer (5'-3')	Reverse Primer (5'-3')
Muc5ac	TCTACTGACTGCACCAACACA	TCCGTCAGTCCACACTTTCG
Muc5b	CTGAAGACCTGTCGGAACCCAA	GCCACACACTTCATCTGGTCCT
P4ha3	TGCAAGTGGAGTACCGCATCAG	CACCTGGAGATACTCTGCGTAG
Bpifa1	GGTCTTGTGCAGAGTCCTGATG	CTTTCACGGCTAAGACTTCTGCA
Bpifb1	CAGGCACTGAAGGACCATGATG	GGATGTTAGCAGAGGTGACCTTC
β-actin	CATTGCTGACAGGATGCAGAAGG	TGCTGGAAGGTGGACAGTGAGG

Table S2. Mortality of chlorhexidine-exposed mice at different concentrations

Dose (%)	Death	Alive	Total	Mortality (%)
0	0	13	13	0
0.125	0	12	12	0
0.25	9	19	28	32.1
0.5	12	16	28	42.9
1	7	3	10	70.0

Table S3. Percentage of inflammatory cells in bronchoalveolar lavage fluid

Group	Macrophages	Lymphocytes	Neutrophils
Control	89.72±1.12	9.51±1.21	0.78±0.31
0.125%	88.35±1.14	10.68±0.65	0.97±0.82
0.25%	85.21±1.42*	13.11±1.21*	1.68±0.53
0.5%	85.61±1.58*	13.54±1.67*	0.85±0.12

*$P < 0.05$， compared with control group.

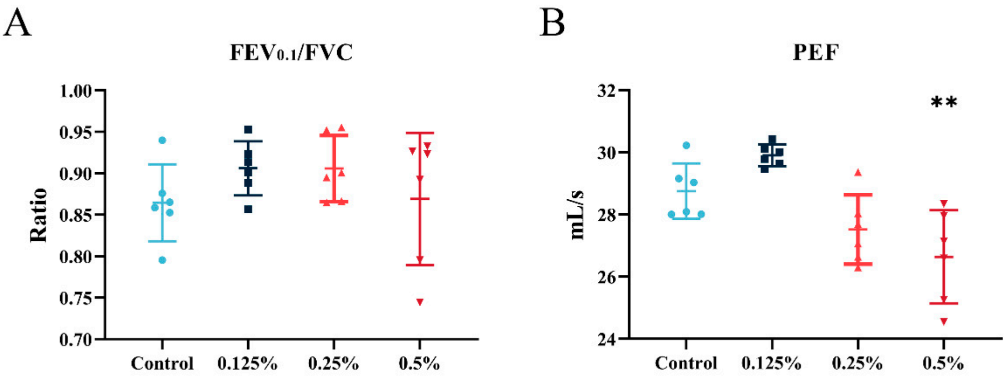

Figure S1. Chlorhexidine decreased pulmonary function in mice. (A) $FVE_{0.1}$/FVC and (B) Peak expiratory flows (PEF) were measured using a NPFE model via the flexiVent FX system. Data were analyzed by one-way ANOVA with Dunnett's post-hoc test (** $P < 0.01$).

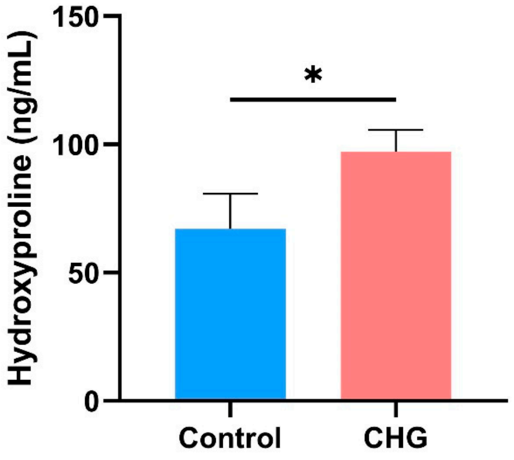

Figure S2. ELISA detection of hydroxyproline in BALF of control and CHG exposure groups. Data are presented as mean ± SD (n = 3, * $P < 0.05$).
